# Peptidomic changes in the milk of water buffaloes (*Bubalus bubalis*) with intramammary infection by non-aureus staphylococci

**DOI:** 10.1038/s41598-022-12297-z

**Published:** 2022-05-19

**Authors:** Maria Filippa Addis, Elisa Margherita Maffioli, Martina Penati, Mariangela Albertini, Valerio Bronzo, Renata Piccinini, Francesco Tangorra, Gabriella Tedeschi, Giovanna Cappelli, Gabriele Di Vuolo, Domenico Vecchio, Esterina De Carlo, Fabrizio Ceciliani

**Affiliations:** 1 Department of Veterinary Medicine and Animal Sciences, Università degli Studi di Milano, Lodi, Italy; 2 CIMAINA, Università degli Studi di Milano, via Celoria 10, Milan, Italy; 3 CRC “Innovation for Well-Beeing and Environment” (I-WE), Università degli Studi di Milano, Milan, Italy; 4 National Reference Center on Hygiene and Technologies of Buffalo Breeding and Production, Istituto Zooprofilattico Sperimentale del Mezzogiorno, Salerno, Italy

**Keywords:** Peptides, Proteomics, Bacteriology

## Abstract

Mastitis by non-aureus staphylococci (NAS) is a significant issue in dairy buffalo farming. In a herd with subclinical NAS mastitis, we identified *Staphylococcus microti* as the predominant species. To assess milk protein integrity and investigate potential disease markers, we characterized 12 NAS-positive and 12 healthy quarter milk samples by shotgun peptidomics combining peptide enrichment and high-performance liquid chromatography/tandem mass spectrometry (LC–MS/MS). We observed significant changes in the milk peptidome. Out of 789 total peptides identified in each group, 49 and 44 were unique or increased in NAS-positive and healthy milk, respectively. In NAS-positive milk, the differential peptides belonged mainly to caseins, followed by milk fat globule membrane proteins (MFGMP) and by the immune defense/antimicrobial proteins osteopontin, lactoperoxidase, and serum amyloid A. In healthy milk, these belonged mainly to MFGMP, followed by caseins. In terms of abundance, peptides from MFGMP and immune defense protein were higher in NAS-positive milk, while peptides from caseins were higher in healthy milk. These findings highlight the impact of NAS on buffalo milk quality and mammary gland health, even when clinical signs are not evident, and underscore the need for clarifying the epidemiology and relevance of the different NAS species in this dairy ruminant.

## Introduction

The water buffalo (*Bubalus bubalis*) is the second most relevant dairy species after the cow (*Bos taurus*)^1^, with over 97 million tons of milk produced each year^2^. Mastitis caused by an intramammary infection (IMI) is one of the diseases with the highest impact on the economic performance and welfare of dairy animals^3^. Water buffaloes are generally regarded as less susceptible to mastitis than cows^4,5^. Still, the real impact of intramammary infections (IMI) may be underestimated due to the higher prevalence of subclinical mastitis and issues with the setting of somatic cell count (SCC) thresholds^5,6^, which need proper implementation for mastitis monitoring within dairy herd improvement programs.

The main etiologic agents of clinical and subclinical IMI in buffalo are staphylococci^5,6^. *S. aureus* is a highly impacting pathogen for clinical severity and ability to spread and persist in the herd, but non-aureus staphylococci (NAS) are most frequently isolated from the milk^6–8^*.* Moreover, milk NAS in water buffalo have been recently reported as a source of antibiotic resistance^9–13^.

The relationship between different NAS and mammary gland health is poorly known. Identification of NAS at the species level is seldom carried out in routine milk bacteriology because of analytical cost issues, combined with the sub-optimal performance of traditional biochemical methods^5,7^. Genotypic identification is also problematic in some cases due to the high similarity between some species^14^. When possible, NAS identification is carried out by matrix-assisted laser desorption/ionization time-of-flight mass spectrometry (MALDI-TOF-MS)^15–17^. Recently, we detected significant changes in the protein composition of buffalo milk with staphylococcal mastitis^8^. In that study, we highlighted the need to clarify the role of the different NAS species in this dairy animal and to further investigate the impact of NAS on buffalo milk quality. Shotgun peptidomics is an approach providing an in-depth perspective on the changes occurring in the peptide profile of many dairy products, adding useful information to the proteomic approach^18^. This method can assess the impact of different conditions by combining the simultaneous identification of thousands of peptides with their quantification in each sample^19^. Therefore, this approach is ideal for quantitatively investigating the differences in the peptidome of milk from healthy animals compared to that from infected udder quarters with mastitis^30^.

In this study, we investigated the impact of NAS IMI on the buffalo milk peptidome with a proteomic analysis pipeline entailing peptide enrichment, high-performance liquid chromatography/tandem mass spectrometry, and bioinformatic analysis, taking into account the causative NAS species and the milk SCC in the definition of the sample groups.

## Results

### Milk somatic cell counts and bacteriology

*Staphylococcus microti* was identified in all the NAS-positive milk samples with MALDI-TOF-MS scores higher than 2.00 but in two cases (1.93 and 1.95, respectively). Three milk samples showed the growth of a second colony type, identified as *Aerococcus viridans* in one sample and *Streptococcus uberis* in two samples. The downstream peptidomic analysis was carried out by classifying the samples according to the combination of the bacteriological and somatic cell count (SCC) information, as detailed in Table 1. The complete data are reported in Supplementary Table 1.

**Table 1:** Somatic cell count (SCC) and bacterial species identified in the quarter milk samples subjected to peptidomic analysis. The last column reports the sample classification according to SCC value and presence of NAS. Complete data are reported in Supplementary Table 1.

N	SCC^1^	NAS^2^ species	CFU/mL^3^	Score^4^	Other bacteria	CFU/mL^3^	Score^4^	Sample group
1	147,000	*Staphylococcus microti*	4000	1.95				NAS-positive
2	290,000	*Staphylococcus microti*	1000	2.07				NAS-positive
3	247,000	*Staphylococcus microti*	2000	1.93				NAS-positive
4	246,000	*Staphylococcus microti*	1000	2.09				NAS-positive
5	242,000	*Staphylococcus microti*	500	2.04	*Aerococcus viridans*	500	2.03	NAS-positive
6	233,000	*Staphylococcus microti*	1000	2.12				NAS-positive
7	211,000	*Staphylococcus microti*	500	2.17				NAS-positive
8	156,000	*Staphylococcus microti*	500	2.16				NAS-positive
9	457,000	*Staphylococcus microti*	2000	2.13				NAS-positive
10	413,000	*Staphylococcus microti*	2000	2.1	*Streptococcus uberis*	2000	2.29	NAS-positive
11	190,000	*Staphylococcus microti*	2000	2.27	*Streptococcus uberis*	2000	2.03	NAS-positive
12	335,000	*Staphylococcus microti*	2000	2.06				NAS-positive
13	30,000	Culture-negative						Healthy
14	21,000	Culture-negative						Healthy
15	20,000	Culture-negative						Healthy
16	50,000	Culture-negative						Healthy
17	34,000	Culture-negative						Healthy
18	24,000	Culture-negative						Healthy
19	51,000	Culture-negative						Healthy
20	48,000	Culture-negative						Healthy
21	36,000	Culture-negative						Healthy
22	73,000	Culture-negative						Healthy
23	54,000	Culture-negative						Healthy
24	27,000	Culture-negative						Healthy

^1^Somatic cell count in cells/mL of milk. ^2^Non-aureus staphylococci. ^3^Colony-forming units per mL of milk. ^4^Log score of the species identification by MALDI-TOF-MS.

### Differential peptidomics

**Figure 1:**
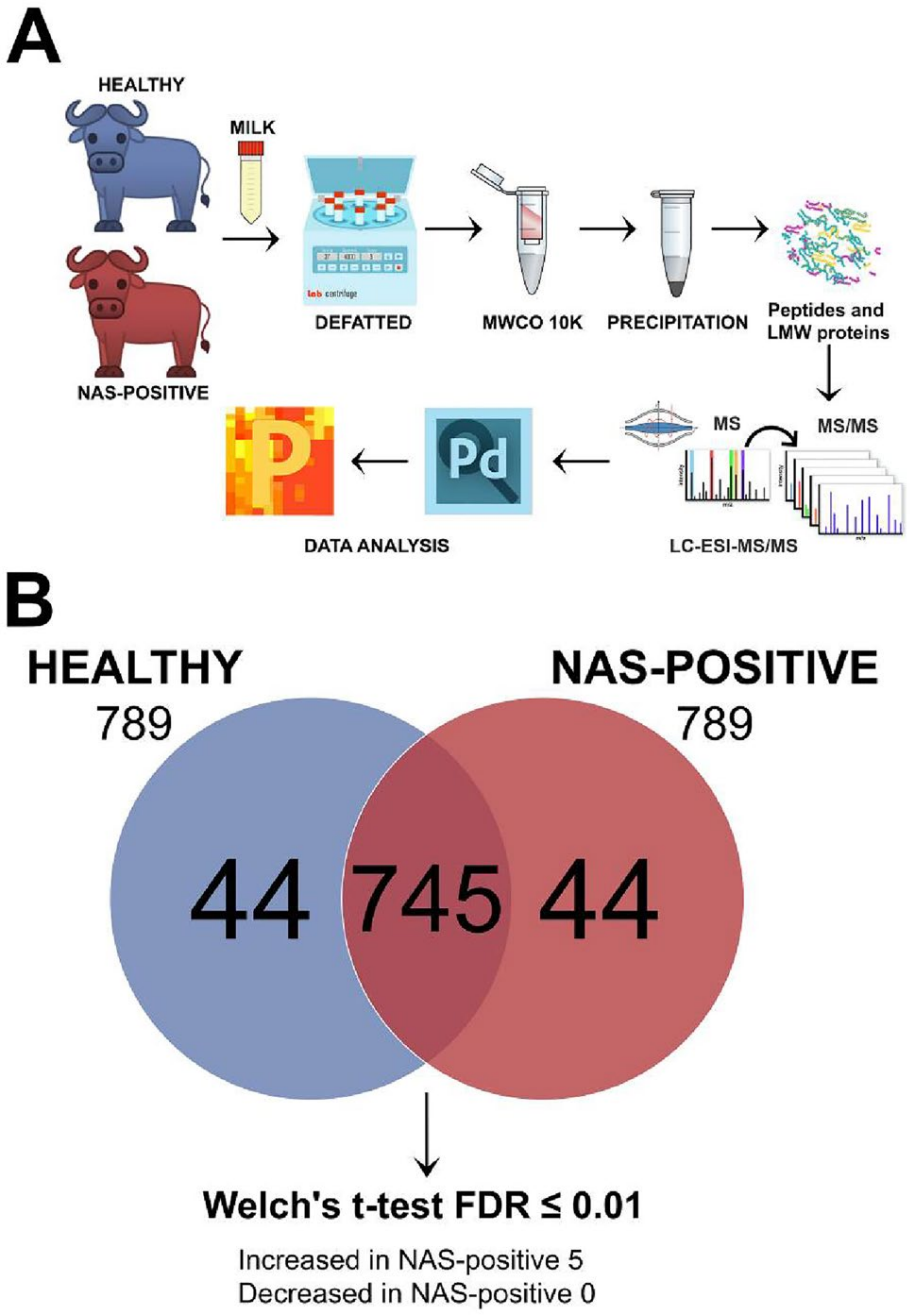
Shotgun label-free quantitative peptidomic analysis. (**A**) Overview of the protocols applied for the analysis of peptides in NAS-positive and healthy milk samples (**B**) Venn diagram of all the peptides identified in milk samples from healthy and NAS-positive buffaloes. Peptides were considered differentially abundant if they were present only in NAS-positiver or Control milk or showed significant Welch *t* test difference (cut-off at 1% permutation-based False Discovery Rate).

The milk samples listed in Table 1 were subjected to a pipeline entailing peptide enrichment, peptide analysis by high-performance liquid chromatography/tandem mass spectrometry (LC–MS/MS), and bioinformatic analysis to identify differential peptides in the two sample groups. The experimental protocol used in this study is schematically summarized in Fig. 1A.

789 and 789 peptides were identified in the NAS-positive and healthy milk samples, respectively, for a total of 833 identified peptides (Fig. 1B).Among the 745 peptides present in both groups, 5 were increased in NAS-positive in comparison to healthy milk (Welch's *t *test: FDR 0.01). No peptides were found decreased in NAS-positive milk. Overall, the analysis identified 49 peptides which were increased (5) or present only in NAS-positive (44), and 44 peptides which were present only in healthy milk (44). Table 2 reports the number of total peptides and the number of unique and differential peptides identified in NAS-positive and healthy milk samples.

**Table 2:** Total and unique peptides identified in the two sample groups by LC–MS/MS and differential analysis. Complete data are reported in Supplementary Table 2.

Sample group	No. of total peptides	No. of unique and differential* peptides
NAS-positive	789	44 + 5*
Healthy	789	44

*Increased in the sample group based on the Welch *t *test with FDR ≤ 0.01.

Table 3 details the sequence of all the unique and differential peptides identified in NAS-positive and healthy milk, their originating protein, and the cell location/function based on the UniProtKB protein knowledge base or scientific literature sources^20–25^.

**Table 3:** Unique and significantly differential* peptides found in NAS-positive and healthy milk with the respective originating protein and its location/function according to the UniProtKB protein knowledge base or referenced literature sources, listed according to the originating protein and to the number of peptides derived from that protein.

	Originating protein	Location/function
**NAS-positive milk**		
IPNSLPQNIPPLTQTPVVVPPFLQPEIMGVSKVKEAMAPKHKEMPFPK	Casein beta	Casein micelle
IPNSLPQNIPPLTQTPVVVPPFLQPEIMGVSKVKEAMAPK		
EELNVPGEIVESLSSSEESITHINKK		
EKFQSEEQQQMEDELQDK		
LTQTPVVVPPFLQPEIMGVSKVKEAMAPKHK		
LLYQEPVLGPVRGPFPI		
FLLYQEPVLGPVRGPFP		
VVPPFLQPEIMGVSKVKEAMAPKHK		
AVPYPQRDMPIQAFLLYQEPVLGPVRGPFPII		
LSLSQSKVLPVPQK		
FLLYQEPVLGPVRGPFPI		
APKHKEMPFPK		
TQTPVVVPPFLQPEIMGVSKVKEAMAPKH		
AMAPKHKEMPFPK		
VVPPFLQPEIMGVSKVKEAMAPK*		
FAWPQYLKTVYQYQKAMKPWTQPK	Casein alpha S2	Casein micelle
NAVPITPTLNREQLSTSEENSKKTVDMESTEVFTK		
DMESTEVFTK		
EQLSTSEENSKKTVDMESTEVITK		
SSEESIISQETYK		
TVYQYQKAMKPWTQPKTNVIPYVRY		
NAVPITPTLNREQLSTSEENSKKT*		
MADEAESLEDLGFKGAHTTQKGHAKARP	Casein alpha S1	Casein micelle
RPKQPIKHQGLPQGVLNENLLRFFVAPFPEVFGKEKV		
KQPIKHQGLPQGVLNENLLRFFVAPFPEVFGK		
FFVAPFPEVFGKEKV		
FVAPFPEVFGKEKV*		
VAPFPEVFGKEKV*		
LPLTKDELEKEAKKVEGFDMVQKPSYYVR	Perilipin 2	MFG membrane^20^
STITGVVDRTKGAVTGSVEK		
INTVLGSRVMQ		
RQPQNQNPKLPLSILKEKHL	Glycosylation-dependent cell adhesion molecule 1	MFG membrane^20^
SSRQPQNQNPKLPLSILKEKHLRN		
ILNEPEDETHLEAQPTDASAQFIRNLQISNEDLSK		
LPVKPTSSGSSEEKQLNNKYPDAVATWLKPDPSQK	Osteopontin	Immune defense^21,22^
PTDIPTIAVFTPFIPTESTNDGRGDSVAYGLKSRSKKF		
RSNVQSPDATEEDFTSHIESEEMHDAPK		
VAPEEHPVLLTEAPLNPK	Actin beta	Cytosol
EITALAPSTMK		
VSPAVFVSREGREQE	Butyrophilin subfamily 1 member A1	MFG membrane^20^
IVDYYEPR	CD109 molecule	Plasma membrane
NVQTEIVNKHNDLRRGVSPPPRNML	Cysteine-rich secretory protein 3	Secreted
DVEKDEKLIRL	DEAD-box helicase 5	Nucleus/cytosol
NRHGSKASADESLALGKPGKEPR	Fibroblast growth factor-binding protein 1	Plasma membrane
PARVLDLGPITR	Pancreatic secretory granule membrane major glycoprotein GP2	MFG membrane^20^
KTTLSSEAPTTQQLSEYFKHAKGQT	Lactoperoxidase	Immune defense
APAGAAIQSRAGEIQ*	Polymeric immunoglobulin receptor	MFG membrane^23^
NPLFEKRPKNF	Ribosomal protein L7a	Cytosol
VISNARETIQGITDPLLKGMTRDQVREDSKADQFANEWGR	Serum amyloid A3 protein	Immune defense^24^
**Healthy milk**		
DVPSERYLGYLEQLLR	Casein alpha S1	Casein micelle
KKYNVPQLEIVPNLAEEQLHSM		
QLEIVPNLAEEQLHSM		
EKVNELSTDIGSESTEDQAMEDIKEQLLR		
STDIGSESTEDQAMEDIK		
KKVEGFDMVQKPSYYVRLG	Perilipin 2	MFG membrane^20^
IHPFAQTQSLVYPFPGPIPKSLPQNIPPLTQTPVVVPPFLQPEIMGVSKVKEAMAPK	Casein beta	Casein micelle
VYPFPGPIPKSLPQNIPPLTQTPVVVPPFLQPEIMGVSKVKEAMAPK		
RELEELNVPGEIVESLSSSEESITHIN		
LYQEPVLGPVRGPFPIIV		
VLPVPQKAVPYPQRDMPIQAFLLYQEPVLGPVRGPFP		
EQEGEEIAEYRGR	Butyrophilin subfamily 1 member A1	MFG membrane^20^
FREKVSPAVFVSREGR		
IPASLFPRLTPWM		
SPAVFVSREGREQEGEEIAEYR		
EEFPSMSESRNPDEEGLFTVR		
NAVPITPTLNREQLSTSEENSKKTVDMESTEVITKKTK	Casein alpha S2	Casein micelle
FPQYLQYLYQGPIVLNPWDQVKR		
KTKLTEEDKNRLN		
LNEINQFYQK		
SSRQPQNQNPKLPLSILKEKHLRNAA	Glycosylation-dependent cell adhesion molecule 1	MFG membrane^20^
PQNQNPKLPLSILKEKHL		
QPQNQNPKLPLSILKEKH		
EQIVIRSSRQPQNQNPKLPLSILKEKHL		
DTIAQAASTTTISDAVSKVKIQVNKAFLDSRT	Lactoperoxidase	Immune defense
DTIAQAASTTTISDAVSKVKIQVNKAFLDSRTRL		
WPELENGQPTSEKYTVKADGEQSAKPEKAKETEKDDTGTPITKIEFVPSH	Sodium-dependent phosphate cotransporter 2B	MFG membrane^20,25^
TPAQFDAEELR	Annexin A1	MFG membrane^20^
TPAQFDAEELRAAM		
KTDLEKDIVSDTSGDFRK	Annexin A2	MFG membrane^20^
ELIDQDARDLYDAGVK		
LSRYPSYGLNYYQQKPVALINNQFLPYPYYAKPAAVRSPAQIL	Casein kappa	Casein micelle
QEQNQEQPIR		
EGVAINPARVLDLGPITR	pancreatic secretory granule membrane major glycoprotein GP2	MFG membrane^20^
NPARVLDLGPITR		
KKPPQQLEWKLNTGRTEAW	Advanced glycosylation end-product specific receptor	Cell membrane
LAVRGEPGDSAAEEAAALTGEWR	Cyclin and CBS domain divalent metal cation transport mediator 3	Cell membrane
EIAEAYETLSDANR	DnaJ Hsp family (Hsp40) member B9	Vesicle membrane
FPALTEGYVGTLHENRQGSSVQAQIR	Protein tyrosine phosphatase receptor type G	Cell membrane
NPQGQSQITDPRQAQSSPPWSY	RUNX family transcription factor 2	Nucleus
YNAPPEVVAAKMEVK	Secretoglobin family 1D member	Secreted
WSGPVGVSWGLR	Sortilin 1	Vesicle membrane
NYLEDGESDGFLR	Transmembrane protein 59	Vesicle membrane
VSTKGKRKPRQEEDEDYREFPQKKHKLYGRKQRPKAQPNPK	Zinc finger protein 512	Nucleus

*Peptides significantly more abundant in the sample group according to the Welch *t* test with FDR ≤ 0.01. *MFG* milk fat globule.

**Figure 2:**
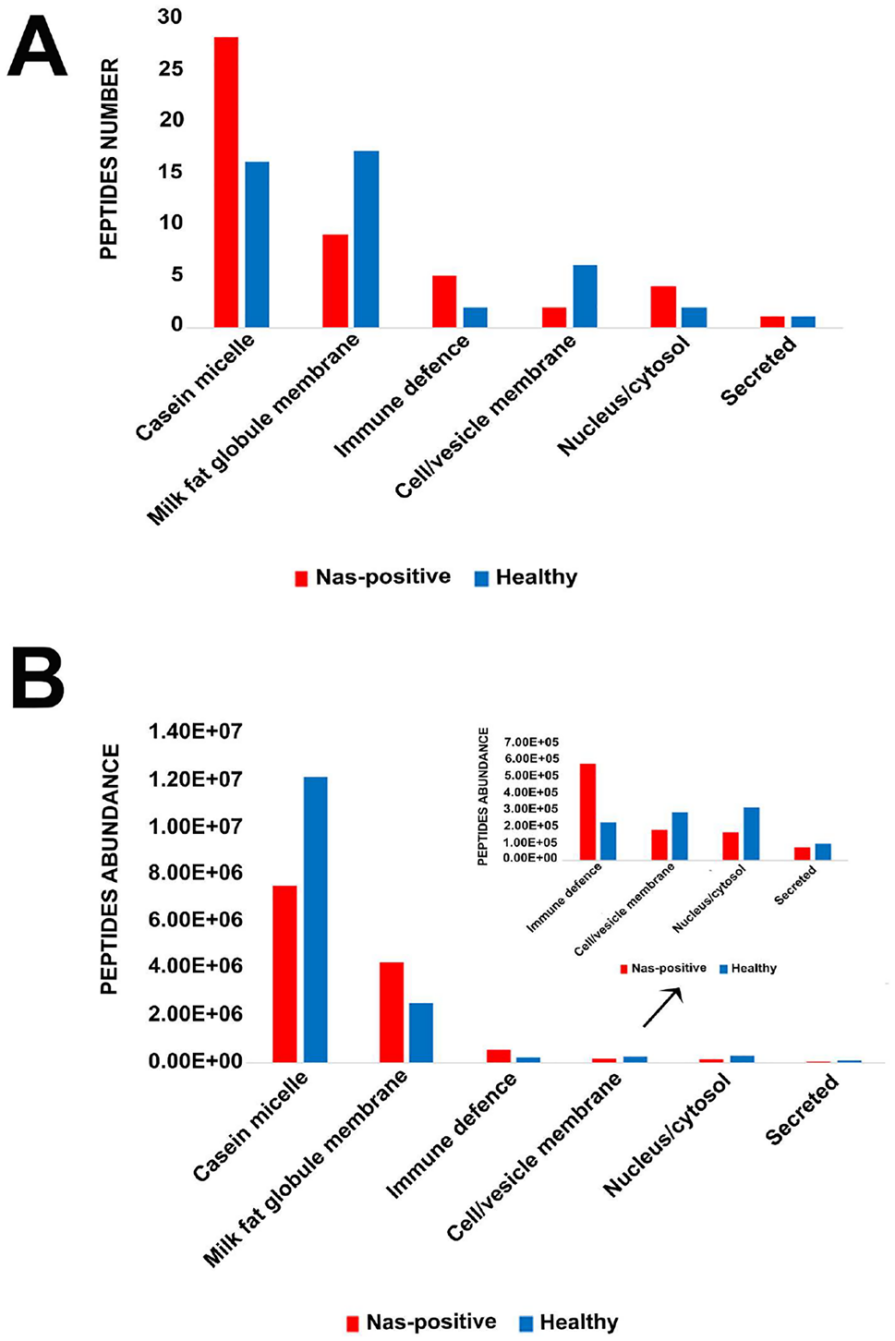
Distribution of the differential peptides identified in NAS-positive (red) and healthy milk (blue) according to the cell location/function of the originating protein. The histograms display the number (**A**) and the abundance (**B**) of peptides in NAS-positive and healthy milk samples, respectively.

Figure 2A,B illustrate the distribution of all unique and differential peptides identified in NAS-positive and healthy milk in terms of number and abundance, respectively, according to the cell location/function of the originating protein and highlight the different nature of the unique and differential peptides identified in the two sample groups. The number of total and differential peptides identified in the two groups was similar, but their nature in terms of originating proteins differed. In NAS-positive milk, 28 of the 49 peptides (57.14%) belonged to caseins, mainly beta-casein (15, 30,61%), followed by alphaS2 (7, 14.29%) and alphaS1 (6, 12.24%), and 9 peptides belonged to proteins of the milk fat globule membrane (MFGMP) (18.37%). Interestingly, 5 peptides belonged to proteins with immune defense/antimicrobial functions (10.20%), namely osteopontin, lactoperoxidase, and serum amyloid A-3. The peptides belonging to proteins with other locations/functions, including cell/vesicle membrane, nucleus/cytosol, and secreted, were 7 out of 49 (14.29%). Conversely, in healthy milk, most unique peptides (17 out of 38,63%) belonged to MFGMP, and 16 (36.36%) belonged to caseins, mainly beta casein (5, 11.36%), alphaS1 (5, 11.36%), alphaS2 (4, 9.09%), and kappa (2, 4.55%). Only 2 unique peptides (4.54%) belonged to inflammatory/immune defense proteins. The remaining 9 unique peptides belonged to proteins with other locations/functions (20.45%).

In terms of their relative abundance, those derived from casein micelle proteins were higher in healthy milk, while those derived from MFGMP were higher in NAS-positive milk. On the other hand, Immune defense proteins were higher in NAS-positive milk also in terms of relative abundance.

**Figure 3:**
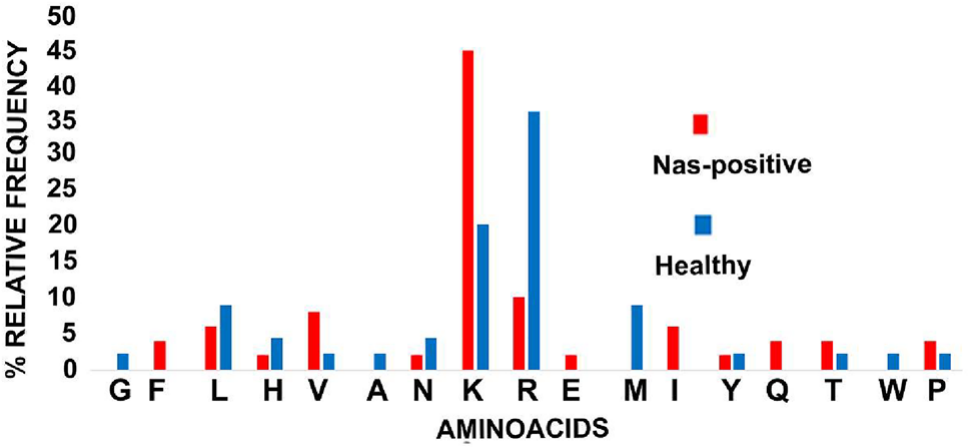
Relative amino acid frequencies at the C-terminus. The figure illustrates the relative distribution of C-terminal amino acids in unique and significantly differential peptides found in NAS-positive (red) and healthy milk (blue), respectively.

The differential peptides were manually analyzed and classified according to their C-terminal amino acid. As shown in Fig. 3, R at the C-term was considerably less frequent (10.2%) in the peptides unique or more abundant in healthy milk. On the other hand, peptides ending especially with K (44.9%), V (8.16%), and F (4.08%) were more frequent in NAS-positive milk.

## Discussion

Based on our findings, the presence of a NAS IMI was associated with changes in the peptide composition of water buffalo milk. The differential peptides identified were derived from proteins with very different functions and localizations. As milk quality and technological properties may be affected, this deserves consideration^26^.

We detected four differential peptides from serum amyloid A (SAA) in NAS-positive animals. SAA is associated with high SCC and mastitis in bovine cows^24,27^, being an acute phase protein^24^ that is overexpressed in milk during mastitis^28,29^. The mammary gland produces a specific form of SAA, the M-SAA3^30,31^, which can be upregulated by *S. aureus* lipoteichoic acid^32^. One differential peptide originating from SAA A-3 (VISNARETIQGITDPLLKGMTRDQVREDSKADQ FANEWGR) was found uniquely in NAS-positive milk, in line with our previous finding of the SAA protein only in the milk of water buffaloes with staphylococcal IMI by shotgun proteomics^8^. Interestingly, in another shotgun peptidomics study, we detected SAA peptides only in cows with NAS IMI^33^. Thus, our detection of SAA only in the milk of animals with NAS IMI further supports its diagnostic potential in the dairy buffalo^34^. Nevertheless, the influence of other physiological variables including parity and stage of lactation on M-SAA levels will have to be assessed^35^.

Three unique and significantly differential peptides originating from osteopontin were found in NAS-positive milk. This is also in line with our previous peptidomic study on bovine cows^33^, although we did not identify the intact protein by proteomics in the water buffalo^8^. Among other biological roles, osteopontin upregulates interferon-gamma and interleukin-12 and downregulates interleukin-10 and plays a role in inducing type I immunity^36^. In cows, osteopontin peptides have been reported in subclinical mastitis^37^ and experimental *Escherichia coli* IMI^21,22^. As also indicated in a recent review on NAS affecting cows, this further indicates that NAS indeed elicit an inflammatory response in the mammary gland, as confirmed by the increased milk SCC. The present finding may support the hypothesis that NAS provide cross-protection against other mastitis pathogens^38^ as components of the mammary gland microbiota^39,40^.

On the other hand, most of the unique peptides found in healthy milk belonged to proteins of the milk fat globule membrane (perilipin 2, butyrophilin, GLYCAM-1, sodium-dependent phosphate cotransporter, annexins, glycoprotein-2)^25,41^, in line with the observations made by shotgun proteomics^8^. The predominance of MFG membrane proteins in healthy milk might be related to the high fat content of buffalo milk, and therefore to the higher abundance of these lipid secretion vesicles compared to cow milk. MFG are an important source of nutraceutical components, including membrane proteins, and the possible influence of NAS IMI on their integrity may deserve further consideration concerning nutritional value, product quality, and technological properties^26^. When looking at the differential distribution of peptides in terms of abundance, we observed that healthy milk was characterized by a higher abundance of casein proteolytic peptides, and NAS-positive milk by a higher abundance of peptides derived from MFG membrane proteins and immune defence proteins. While the first finding might be influenced by the higher abundance of caseins in healthy vs infected milk, the second finding further highlights the impact of NAS IMI on integrity and abundance of MFG membrane proteins and immune defence proteins, respectively, reinforcing the above considerations^8^.

The distribution of unique and differential peptides based on their C-terminal aminoacid showed a higher frequency of peptides ending with R in healthy milk as opposite to peptides ending especially with K, V, and F, in NAS-positive milk, in line with the observations made by our previous peptidomic work in bovine cows^33^. According to the MEROPS database, plasmin generates peptides ending with R and K at the C-term, while elastase, cathepsin D and cathepsin G generate peptides ending with V and F at their C-term^42^. Our results suggest a more intense proteolytic activity by plasmin and endogenous proteases released by inflammatory cells in NAS-positive milk.

The impact of NAS IMI on the buffalo milk peptidome was less intense than observed in cows in our recent work^33^. However, as mentioned above, many findings were consistent including the presence in NAS-positive milk of peptides derived from osteopontin and SAA, and the different frequency of C-terminal aminoacids in the proteolytic peptides of the two sample groups^33^.

Concerning the etiologic agent, the identification of *S. microti* as the predominant species in the milk of water buffaloes with subclinical mastitis is noteworthy as only one study reported its association with mastitis in bovine cows^43^. *S. microti* is closely associated with *S. rostri* and *S. muscae*, and it has been first isolated from *Microtus arvalis*, the common vole. Since its description, it has been isolated from rodents/insectivores and a female sandfly^43^. Therefore, the role of animal vectors might be relevant in this case. Adding to anatomical and physiological characteristics, important differences characterize bubaline cows and bovine cows in terms of animal management, farming practices (housing, feeding, bedding, milking routine), environmental temperature and humidity, and presence of water ponds, and consequently contact with different microbial reservoirs including wild and domestic animals. This may lead to mammary gland exposure and colonization by other NAS species than the bovine dairy cows, as well as to different bacterial loads in the farm environment, and should be carefully considered.

## Methods

### Animals and milk samples

The study was carried out on quarter milk samples collected from a commercial water buffalo dairy farm located in Campania, Southern Italy, with an increased bulk tank somatic cell count related to NAS IMI. The farm maintained the milking buffaloes in free-stall barns with deep-bedded cubicles with straw. All the animals were fed with a balanced Total Mixed Ration in feed alleys with headlocks. Lactating cows were milked twice a day in a double-10 herringbone parlour milking. The farm was free of brucellosis and paratuberculosis. All the milk samples used for this study were collected within the frame of a diagnostic routine visit for monitoring the health status of the herd. This practice is approved by the Ethical Committee of the University of Milan (Comitato Etico 15.02.16 Parere numero 2/16) “allowing the use, under informed consent of the owners, of the residual volume of samples for studies on metabolic biomarkers”. All methods and procedures were performed in accordance with the relevant institutional guidelines and regulations. The methods described and the results reported were compliant with the ARRIVE guidelines for reporting animal research^44^.

Milk samples were processed as indicated by the National Mastitis Council^45^. Before sampling, teats were cleaned with a pre-dipping foam containing lactic acid, and the apex was scrubbed and disinfected with alcohol. The first streams of milk were discharged, and 20 mL was collected aseptically from each quarter into sterile vials. The milk samples were kept at 4 °C until they reached the laboratory (within the day) at the IZS in Portici for bacteriological assays and somatic cell count.

### Bacteriological analysis and somatic cell count

Bacteriological cultures were performed according to the National Mastitis Council^45^. Briefly, 10 µL of milk was spread on blood agar (Trypticase Soy Agar with 5% defibrinated sheep blood) and incubated at 37 °C for 24 h in aerobic conditions. For a preliminary identification of the etiological agent, Gram staining, catalase, and coagulase tests were performed on the colonies observed for all positive cultures, as part of the routine milk analysis. The SCC was measured in milk samples using the Fossomatic (Foss) instrument following the UNI EN ISO 13366-2: 2007 standard and expressed as the number of cells per mL of milk. Two equally populated sample groups (12 samples per group) were defined based on the combined results: NAS-positive (no more than two colony types, bacterial count per colony ≥ 500 colony-forming units per mL (CFU/mL), SCC ≥ 100,000 cells/mL) and healthy (no growth, SCC < 100,000 cells/mL).

### MALDI-TOF-MS for bacterial identification

Milk samples growing coagulase-negative staphylococci at the routine milk analysis were sent frozen to the milk quality laboratory at the University of Milan for microbial identification by MALDI-TOF-MS. There, milk samples were thawed at room temperature, and 100 µL of milk was spread on blood agar half-plates to obtain bacterial colonies for MALDI-TOF-MS identification. After incubation for 24 h at 37 °C, the plates were examined for microbial growth. When no more than two different colony types were present, these were counted and assessed by MALDI-TOF-MS for microbial identification as described previously^46^, with minor modifications. One colony was deposited in duplicate on the target plate using a toothpick, overlaid with 1 µL of α-cyano-4-hydroxycinnamic acid (Bruker Daltonik GmbH, Bremen, Germany) and left to dry. The spectra were acquired with a microFlex™ mass spectrometer (Bruker Daltonik GmbH) in the positive mode, including two spots of Bacterial Test Standard (Bruker Daltonik GmbH) for calibration in each target plate. The obtained spectra were interpreted against the MBT Compass^®^ 4.1 database. Log scores of ≥ 1.7 and ≥ 2.0 were the thresholds for the genus and species level identification, respectively.

#### Milk sample preparation for peptidomic analysis

The milk samples were processed for peptidomic analysis as described previously^33^. Briefly, milk was defatted by centrifugation and processed for the depletion of high-molecular-weight proteins on centrifugal filters. The filtrate was precipitated, and peptides were dried, dissolved in 1% (v/v) formic acid and desalted before MS analysis.

#### Tandem mass spectrometry analysis of peptides

Tandem mass spectrometry analysis of peptides was carried out with duplicate runs for each sample as described previously^33^. Briefly, LC-ESI-MS/MS analysis was performed on a Dionex UltiMate 3000 directly connected to an Orbitrap Fusion Tribrid Mass Spectrometer (Thermo Fisher Scientific) by a nanoelectrospray ion source. Peptide mixtures were enriched on 75 μmID × 150 mm Acclaim PepMap RSLC C18 column and separated employing the following LC gradient: 4% ACN in 0.1% formic acid for 3 min, 4–28% ACN in 0.1% formic acid for 130 min, 28–40% ACN in 0.1% formic acid for 20 min, 40–95% ACN in 0.1% formic for 2 min and 95–4% ACN in 0.1% formic acid for 3 min at a flow rate of 0.3 μL/min. MS spectra of eluting peptides were collected over an m/z range of 375–1500 using a resolution setting of 120,000, operating in the data-dependent mode to automatically alternate between Orbitrap-MS and Orbitrap-MS/MSacquisition. HCD MS/MS spectra were collected for the 20 most abundant ions in each MS scan using a normalized collision energy of 30%, and an isolation window of 1.7 m/z. Rejection of + 1 and unassigned charge states were enabled.

#### Database search, peptide identification, and differential analysis

Raw label-free MS/MS files from Thermo Xcalibur software (version 4.1) were analyzed using Proteome Discoverer software (version 2.2, Thermo Fisher Scientific)^47^ and searched with the Sequest algorithm against the proteome of Bubalus bubalis from NCBI 01-08-2019 and Staphylococcus from Uniprot 18-06-2019. Only peptides with high FDR confidence were considered (FDR 0.01 strict, FDR 0.05 relaxed) to remove false-positive matches. The assigned peptides are filtered by minimal peptide length (6 amino acids) and m/z accuracy (8 ppm). The quality of a match between sequence and observed peaks was provided by a high cross-correlation score (≥ 1.5). PSM confidence was set to High.

Unspecific digestion was chosen, and neither fixed nor variable modifications were set. The resulting peptides and protein hits were further screened accepting only those hits listed as high confidence and with an Xcorr ≥ 1.5. Only peptides present and quantified in 66,6% of the repeats were positively identified and used for statistical analysis. Peptides were considered increased or decreased if they showed a significant Welch *t *test difference (cut-off at 1% permutation-based FDR) or if they were present with a frequency ≥ 66,6% in either NAS-positive or healthy milk group but less than 66,6% in the other group^48^. Statistical analysis was performed using the Perseus software (version 1.5.5.3, http://www.biochem.mpg.de/mann/tools/). Peptide sequences were analyzed manually for C-terminal amino acids. The potential proteases generating the cuts were classified based on the MEROPS database^42^ by evaluating the specificities of the main proteases generating the cuts^49^. The protein functions reported in Table 3 were retrieved from the UniProtKB protein knowledgebase (http://www.uniprot.org).

## Supplementary Information

Supplementary Table 1.

Supplementary Table 2.

## Data Availability

The data have been deposited to the ProteomeXchange with identifier PXD028793.
