# Supplementary material for: Peptidomic changes in the milk of water buffaloes (Bubalus bubalis) with intramammary infection by non-aureus staphylococci
Source: Sci Rep. 2022 May 19;12:8371. doi: 10.1038/s41598-022-12297-z (PMC9120474; doi:10.1038/s41598-022-12297-z)
Supplement: Supplementary file 1 — Supplementary Table 1. [file 41598_2022_12297_MOESM1_ESM.pdf]

Supplementary Table 1. Details on milk samples and analytical groups defined for differential peptidomics.

Parity	DIM	Cow n.	Q	SCC	NAS ID	Score	CFU/mL	Other ID	Score	CFU/mL	Group	Notes
2	132	61	FR	147000	*Staphylococcus microti*	1.95	4000				NAS-positive	
2	114	65	FR	290000	*Staphylococcus microti*	2.07	1000				NAS-positive	
2	114	65	FL	247000	*Staphylococcus microti*	1.93	2000				NAS-positive	
6	144	719	RL	246000	*Staphylococcus microti*	2.09	1000				NAS-positive	
6	128	754	RR	242000	*Staphylococcus microti*	2.04	500	*Aerococcus viridans*	2.03		NAS-positive	
5	113	787	RR	233000	*Staphylococcus microti*	2.12	1000				NAS-positive	
5	113	787	FL	211000	*Staphylococcus microti*	2.17	500				NAS-positive	
5	113	787	FR	156000	*Staphylococcus microti*	2.16	500				NAS-positive	
5	204	829	FR	457000	*Staphylococcus microti*	2.13	2000				NAS-positive	
5	204	829	RL	413000	*Staphylococcus microti*	2.1	2000	*Streptococcus uberis*	2.29		NAS-positive	
5	204	829	RR	190000	*Staphylococcus microti*	2.27	2000	*Streptococcus uberis*	2.03		NAS-positive	
4	122	884	RL	335000	*Staphylococcus microti*	2.06	2000				NAS-positive	
2	90	41	FR	30000							Healthy	
2	90	41	RR	21000							Healthy	
2	90	41	FL	20000							Healthy	
2	103	59	FL	50000							Healthy	
11	91	93	FL	34000							Healthy	
11	91	93	RR	24000							Healthy	
6	144	719	FL	51000							Healthy	
5	120	746	RL	48000							Healthy	
5	120	746	FR	36000							Healthy	
5	113	787	RL	73000							Healthy	
4	122	884	RR	54000							Healthy	
3	87	993	FL	27000							Healthy	

DIM: days in milk. Q: quarter. SCC: somatic cell count in cells/mL of milk. NAS ID: identification of staphylococcal colonies MALDI-TOF-MS. Score: Log score obtained by MALDI-TOF-MS. CFU/mL: colony-forming units per mL of milk. Second ID: identification of non-staphylococcal colonies by MALDI-TOF-MS.
